# Utilisation of Targeted Nanoparticle Photosensitiser Drug Delivery Systems for the Enhancement of Photodynamic Therapy

**DOI:** 10.3390/molecules23102628

**Published:** 2018-10-13

**Authors:** Cherie Ann Kruger, Heidi Abrahamse

**Affiliations:** Laser Research Centre, Faculty of Health Sciences, University of Johannesburg, Johannesburg, Doornfontein 2001, South Africa; cherier@uj.ac.za

**Keywords:** photodynamic therapy, cancer, nanotechnology, targeted drug delivery systems

## Abstract

The cancer incidence world-wide has caused an increase in the demand for effective forms of treatment. One unconventional form of treatment for cancer is photodynamic therapy (PDT). PDT has 3 fundamental factors, namely a photosensitiser (PS) drug, light and oxygen. When a PS drug is administered to a patient, it can either passively or actively accumulate within a tumour site and once exposed to a specific wavelength of light, it is excited to produce reactive oxygen species (ROS), resulting in tumour destruction. However, the efficacy of ROS generation for tumour damage is highly dependent on the uptake of the PS in tumour cells. Thus, PS selective/targeted uptake and delivery in tumour cells is a crucial factor in PDT cancer drug absorption studies. Generally, within non-targeted drug delivery mechanisms, only minor amounts of PS are able to passively accumulate in tumour sites (due to the enhanced permeability and retention (EPR) effect) and the remainder distributes into healthy tissues, causing unwanted side effects and poor treatment prognosis. Thus, to improve the efficacy of PDT cancer treatment, research is currently focused on the development of specific receptor-based PS-nanocarrier platform drugs, which promote the active uptake and absorption of PS drugs in tumour sites only, avoiding unwanted side effects, as well as treatment enhancement. Therefore, the aim of this review paper is to focus on current actively targeted or passively delivered PS nanoparticle drug delivery systems, that have been previously investigated for the PDT treatment of cancer and so to deduce their overall efficacy and recent advancements.

## 1. Introduction

Currently, cancer is the second leading cause of death after stroke and heart disease and so represents a major health concern worldwide [1,2]. While improved diagnostic and conventional treatment measures have helped decrease the incidence rates of some cancers such as colorectal and prostate, the current death rates from liver, pancreas, cervical, breast and lung cancers are still on the rise [3,4].

The main issue when treating cancer patients with conventional therapies such as chemotherapy or radiation, is that these forms of treatment tend to have a low selectivity for cancer cells and so are required to be administered in high toxic drug loads to be considered effective [5]. These high toxic drug loads also tend to affect normal body cells as well, often inducing severe unwanted side effects when patients undergo these forms of treatment [5]. Moreover, additional obstacles that most conventional cancer treatments face arise from tumour heterogeneity, drug resistance and systematic toxicities [6].

Thus, the current worldwide spread and rise in cancer incidence and mortality, with the difficulties conventional treatments face, have placed a high demand on research and drug developers for more effective forms of treatment [6]. 

## 2. Photodynamic Therapy

**Figure 1:**
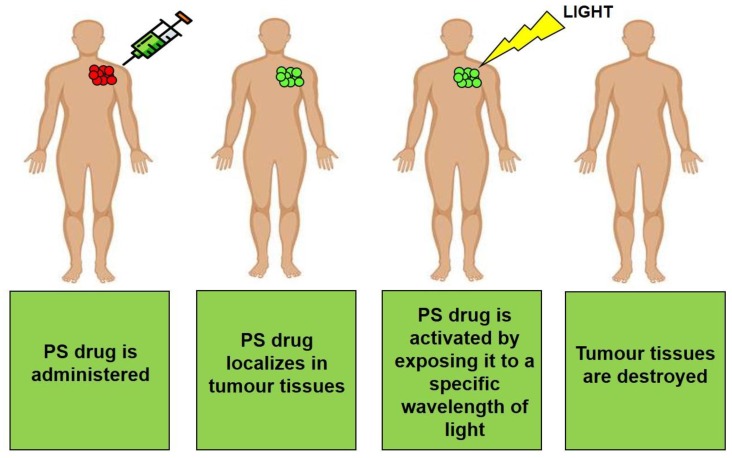
PDT treatment of cancer. The PS drug is administered to a patient and via the bloodstream, it is transported to the tumour site, whereby it localises in tumour cells. Laser light is then applied to this site, whereby it penetrates the skin and activates the PS. The PS then undergoes a photoreaction to produce ROS and/or singlet oxygen, which in turn induces cytotoxic cell death in tumour tissues.

Photodynamic therapy (PDT) is a promising unconventional treatment method, which has been used for the control of a variety of cancers [7]. PDT is a synchronised process, which first requires the administration of a photosensitiser (PS) drug, either topically or intravenously to a patient, dependent on the location of a tumour (Figure 1) [7].

The PS drug compound is then passively or actively absorbed (dependent on drug delivery mechanism) by either cancer cells or tissues [8]. To a certain extent an advantage of PDT is that it can achieve discriminate tumour cell destruction and so induce only slight damage in healthy tissues [8]. This is because PS drugs tend to preferentially localise in diseased tissue via the enhanced permeability retention—EPR passive effect [8]. However, current research is working towards improving this form of passive PS drug uptake to be a more specific and targeted PS delivery in cancer cells only, so that photosensitivity, localised healthy tissue destruction and other additional unwanted side effects can possibly be eliminated [9].

**Figure 2:**
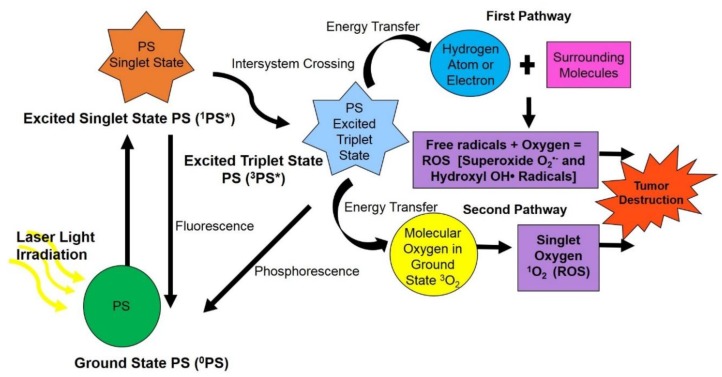
Photodynamic therapy photochemical and photophysical mechanism of photosensitiser drug activation in tumour cells at a specific wavelength of light leading to transfer of oxygen molecules or other substrates in surrounding areas, generating cytotoxic ROS, which triggers apoptotic or necrotic forms of cell death and so tumour cells are destroyed.

Once the PS has successfully localised in targeted tumours, it is then activated by exposing it to a specific wavelength of light (Figure 2) [9]. The PS then absorbs these light photons and so becomes excited and stimulated from a ground state to a higher level of energy, known as a singlet state [10]. Then through a mechanism known as intersystem crossing (which results in a change in the spin of an electron) an excited singlet state PS can then convert into a triplet state PS [8]. The triplet state PS then interacts with surrounding molecules in tumours and so produces reactive oxygen species (ROS) or highly reactive singlet oxygen (^1^O_2_) species via two different pathways [8]. Within the first pathway either a hydrogen atom or electron is transferred from the excited triplet PS to surrounding substrates, causing free radicals to be produced [11]. These free radicals then react with oxygen to form ROS, such as superoxide and hydroxyl radicals. In the second pathway energy is transferred from the excited triplet state PS and ground state molecular oxygen (^3^O_2_), resulting in the formation of highly reactive singlet oxygen (^1^O_2_) species [12]. These final resulting ROS and singlet oxygen species are cytotoxic and so trigger apoptotic, necrotic or autophagy-associated cell death mechanisms in tumour cells via oxidation (Figure 2) [6,7,8,9,10,11]. Both PDT pathway reactions may occur simultaneously, however PSs generally favour the first pathway of ROS generation followed by apoptotic cell death within in vitro anti-cancer PDT [12]. Moreover, the ratio between these pathway processes often depends on the type of PS used, sub-cellular localisation of the PS, as well as the concentration of molecular oxygen within the tumour’s cellular microenvironment [13].

It must be noted that in the absence of an external photo-activating light source, PS drugs remain minimally toxic in the body [9]. Therefore, PDT can provide an alternative treatment method to assist in the eradication of target cancer cells/tissues, while avoiding systematic toxicity and unwanted side effects when compared to conventional therapies (which affect the entire body and not just localised healthy cells) [9], however PS drug delivery mechanisms still require optimization [4].

## 3. Photosensitisers

Photosensitisers (PSs) are chromophore-containing compounds, which are either natural or synthetic in chemical composition [14,15]. PSs have the ability to absorb light at a particular wavelength and so generate cytotoxic ROS, which in turn allows PDT treatments to induce chemical or physical damage in target cancer tissues [7]. However, within PDT applications it is important for PS drugs to have high molar absorption coefficient within the PDT therapeutic red region of the visible light spectrum (650–780 nm), as to ensure minimal patient photosensitivity before PS excitation, as well as to avoid light absorption by other endogenous human body pigments [13,16]. Moreover, these particular wavelength parameters also ensure maximum light absorption for PS excitation and ROS generation, as well as optimal tissue penetration at targeted tumour sites, to warrant effective PDT cancer treatments [10,14].

Commonly, PSs are classified into three groups according to their functional capabilities namely; first-, second- and third- generation [10]. Examples of first-generation PS drugs include; haematoporphyrin derivatives, they are stable; however, they tend to induce photosensitivity in patients and have a poor light tissue depth excitation range [11,14]. Second-generation PSs tend to have a better PDT efficacy as they have less side effects with far deeper tissue laser light excitation ranges [11,14]. Examples of second-generation PSs include; phthalocyanines, benzoporphyrins, purpurins, hypercin and chlorines [11,14,17]. Third-generation PSs consist of second-generation PSs which have bound to passive targeting nanoparticles (NPs) or active targeting agents (i.e., aptamers, peptides etc.) and so tend to report enhanced uptake and the best PDT treatment outcomes in cancer patients [10,14]. 

Currently, examples of clinically approved first- and second-generation PS in oncology include; Porfimer sodium (Photofrin), 5-Aminolevulinic acid (Levulan), Methyl aminolevulinate (Metvixia), Meta tetra(hydroxyphenyl) chlorin (Foscan), N-aspartyl chlorin e6 (NPe6, Laserphyrin), Benzoporphyrin derivative monoacid ring A (Visudyne) and N-hexyl ester of ALA (Cysview) [14,15,17]. Whereas, examples of first- and second-generation PSs that are currently under clinical trials include; Hypocrellin A, Pheophorbide-a, Chlorin e6, Methylene Blue, Hypericin, Phthalocyanine, Rose Bengal, HPPH: 2-(1-Hexyl-oxyethyl)-2-devinyl pyropheophorbide-alpha [14,16,17]. In relation to third-generation PSs, to date, none have received clinical approval for PDT cancer treatments and so remain an important area of research.

Lastly, research is beginning to focus in the development of fourth-generation PS (i.e., a second-generation PS encapsulated in a NP delivery system—making it a third-generation PS, with a co-encapsulated small-molecular inhibitor capable of blocking tumour survival pathways post PDT treatment in order to improve its overall efficacy in clinical settings and so halt possible tumour reoccurrence [18,19]. At this stage, combination treatments with respect to PDT and inhibitors in clinical setting are limited to the treatment of macular degeneration, in which case vascular endothelial growth factors (VEGFs) inhibitors are employed to deter neovascularization of tumours [18,19]. 

## 4. Photodynamic Therapy Challenges

Despite the many positive features of PDT cancer therapy, this form of treatment is still not always fully adapted in clinical settings [7]. Most of these PDT clinical setting drawbacks are due to some of the inherent properties first and second-generation PS drugs have in relation to their solubility, mode of delivery and targeted tumour tissue selectivity [9,16]. 

In order to ensure the overall efficacy of PDT in terms of inducing complete cell death and overall tumour destruction, maximum levels of ROS generation are required and this is highly dependent on the uptake and concentration level of a PS in cancer cells [10]. 

Generally, non-targeted conventional PS drug delivery mechanisms have a poor PDT clinical prognosis, since only minor amounts of the PS drugs are able to passively accumulate in tumour sites (due to EPR), limiting the overall effectiveness of PDT [8,15]. The remainder of the photosynthetic drug either distributes into healthy tissues (causing unwanted side effects—patient photosensitivity or localised healthy tissue damage) or is destroyed by the bodies’ immunological barriers [10]. Moreover, another common issue in clinical settings is that most second and third generation PS drugs are hydrophobic in nature and so have a limited solubility in water, causing them to aggregate during administration, decreasing ROS generation [9]. 

Additionally, since ROS have only a short half-life, only cells that are close to the proximal area of ROS generation i.e., PS localisation, are directly affected by PDT. Moreover, the radius of action of singlet oxygen is very small ≤0.02 µm [13]. Thus, the overall extent of PDT induced cytotoxicity and photodamage is highly dependent on a PSs bioavailabity, as well as its extracellular and intracellular localisation [12]. Moreover, most PSs have shortcomings such as poor water solubility, bioavailability, biodistribution, and target specificity [12]. Nevertheless, NP PS drug carriers are now being investigated, as to ensure PS drug aqueous dispersibility, with improved targeted delivery and concentrated sub-cellular localisation in tumours mitochondria, lysosomes, endoplasmic reticulum, plasma membrane, etc., which play a major role cell death, for more effective PDT cancer treatment outcomes [12,13]. 

Thus, PS drug selective/targeted uptake and active delivery in tumour cells is a crucial factor in PDT cancer drug absorption studies in order to improve the efficacy of PDT cancer treatment. Therefore, current research is focused on developing effective third generation NP drug delivery systems which incorporate second generation PSs [11,16]. These targeted drug delivery approach should more effectively solubilise and traffic PS within the human body to cross cellular plasma membranes and so actively target, localise and accumulate directly in tumour cells/tissues only, resulting in minimal damage and toxicity to normal tissues, as well as to encourage maximum ROS generation within sub-cellular localised tumour cells for PDT treatment enhancement [11,12]. 

## 5. Nanoparticles for Enhanced Passive or Active Photosensitiser Drug Delivery

Recently, PDT research has reported the combination of PS drugs with NPs since they can overcome some of the limitations conventional PS drug delivery methods experience in clinical settings [20]. 

**Figure 3:**
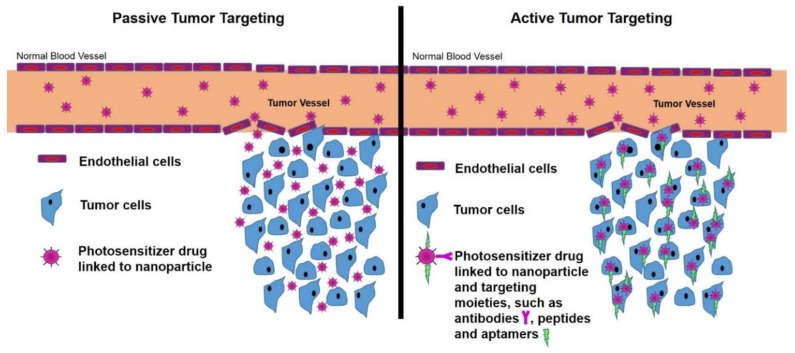
Passive and active forms of photosensitiser (PS) nano-drug tumour targeting and delivery strategies used for the PDT treatment of cancer.

NPs have hydrophilic properties and so when PS drugs are combined with NPs, this significantly enhances their overall solubility and so increases their passive cellular uptake due to the EPR effect [11]. This is because the EPR effect allows NP drug carriers to enter tiny spaces between tumour cells, suppressing lymphatic filtration and so the PS drug uptake in tumour cells is increased (Figure 3) [11]. Additionally, NPs tend to mimic biological molecules, thus when combined with therapeutic drugs such as PSs, the passive cellular tumour uptake of the drug is enhanced [14]. This is due to the fact that PS NP drug carrying systems go by unnoticed by immune system barriers and so remain unharmed by various immune components, allowing for effective PS drug delivery and cellular uptake in tumours [14].

Moreover, NPs are easy to synthesise, have the ability to support high loading volumes of therapeutic drugs (due to there are-to-volume ratios), have a small size (so easily accumulate in cells) and their surface chemistry is simple allowing for possible functionalisation [8]. In a NP drug delivery-based approach, a PS is either encapsulated or immobilised on the NPs surface using covalent or non-covalent interactions to form a nanophotosensitiser (NPPS) [15,20]. Thus, the utilisation of NPs within cancer PDT therapy as effective PS drug delivery systems in research is fast becoming popular [14].

However, since the ultimate goal of PDT is to selectively kill tumour cells with minimal collateral damage to surrounding normal healthy tissues, actively functionalised NPPS drug delivery mechanisms are the current hot topic of research. Thus, in order to improve tumour PS drug uptake selectively and sub-cellular localisation, research studies have been conducted in order to further functionalise NPPS drug delivery systems by linking specific active targeting moieties (biomolecules or ligands) such as antibodies, peptides or aptamers to their surface (Figure 3) [15,20]. These moieties have a specific affinity for specific receptors, which are only overexpressed on the tumour cells (direct active targeting) and their vasculature (indirect active targeting), but not on normal cells [16]. This surface functionalisation of NPPS drug delivery systems facilitates a more effective, specific and active accumulation and sub-cellular of PSs in tumour tissues or cells (via receptor mediated endocytosis—RME) and so overall increases the efficacy of PDT with decremental damage to normal healthy tissues (Figure 3) [9,15,16,17,20].

## 6. Nanoparticle Platforms for Active or Passive Photosensitiser Drug Delivery

To date, many different organic and inorganic nano-platforms have been studied for efficient and targeted PS drug delivery; since they assist in overcoming some of the drawbacks associated with the stability and physiological conditions conventional PS drug delivery face and so enhance the efficacy of PDT [5,20]. 

Organic NPs are solid particles composed of organic compounds such as lipids, protein, polysaccharides or polymers [21]. Examples of organic NP platforms for PDT include liposomes and polymeric NPs (e.g., Albumin, Chitosan, Hyaluronic acid, polymeric micelles, hydrogels, dendrimers, hyperbranched polymers and biodegradable polymers), as well as protein-based NPs (e.g., amino acids, peptides, albumin, gelatin, collagen, silk) (Table 1) [14,22]. These NPs bear the advantage of having low toxicity, as well as mostly improve the solubility of PS drugs and their passive or active accumulation within the target tumour site [14]. PS are generally strategically encapsulated in these types of flexible and versatile NP platforms, for achieving safe and controlled PS drug delivery [14,21].

Inorganic NPs are of metal oxide or metallic composition that normally consist of an inner inorganic core and an outer organic shell, which stabilises the particle in biological environments [14,15]. Moreover, the surface of inorganic NPs can easily be functionalised with various biomolecules for actively selective PS drug targeting of tumorous tissues [16,23,24]. Inorganic NPs have several advantages over organic NPs, including high stability, precise control over shape and size, as well as tuneable optical properties [20,24]. Generally, PSs drugs are incorporated by either physical absorption or covalent attachment onto the reactive surface groups of inorganic NPs [15,23]. However, this PS drug incorporation is highly dependent on the chemical nature of the PS and porosity of the NP (if very porous PS can be physically encased in it, to protect it from degradation) [14,16]. Examples of inorganic biocompatible NP PS drug-based delivery systems include ceramic (silica), quantum dots, magnetic, metallic (gold, silver, zinc or titanium dioxide) and carbon based (fullerene, carbon nanotube and graphene oxide) [14,21,25,26] (Table 1).

Overall, NPs offer a versatile platform for PDT drug delivery by either passively delivering or actively targeting PS in tumour cells, as well offering PDT additional advantages such as enhanced light penetration [16]. 

However, the mode of loading/binding of a PS drug onto a NP vehicle does play a very important role in terms of ensuring an effective dose reaches tumour sites [11,35,36]. NP platforms can either have a PS drug physically entrapped and so loaded within the vehicle core (e.g., micelles) or chemically conjugated/bound onto its NPs surface (e.g., gold NPs) [6,36]. Generally, PS drugs that are entrapped or loaded within NPs drug delivery cores, show better clinical absorption in tumour sites than PS drugs, which are bound onto a NPs surface [6,36]. This is because NP surface absorbed PS drug molecules are sometimes chemically or physically desorbed by in vivo environments, leading to premature drug loss even before a target site is reached [5,10,11]. However, PS drugs, which are physically entrapped in NP cores, often report enhanced delivery, since this encapsulation protects the PS drug from external in vivo factors and so allows it to reach and concentrate in a target site [36,37]. Once a NP encapsulated PS concentrates at a target site it can become photo activated, and so react with cellular oxygen to generate ROS, initiating its release and cytotoxic nature [5,11,14]. Also, another important aspect that researchers need to aware of is that when choosing a NP platform, one needs to ensure that it can hold or has efficient PS drug loading to induce effective PDT activity [16,36,38]. However, the chosen NP platform should not be overloaded with PS drugs, as this can cause aggregation or self-quenching and so reduce PDT efficacy [16,36,38]. 

Furthermore, NPs may be coated with a PEG shielding outer layer that allows for stability of the drug delivery system, as well as provides biocompatibility and so allows NP PSs to have longer circulation times in the body [20,36]. Moreover, NP drug delivery systems can be up-converted and so provide a supplementary advantage by converting low energy radiation to high-energy emission, thereby further facilitating the PDT destruction process in deep-seated tumours [5,14,21]. Likewise, biocompatible functionalised magnetic NPs, such as superparamagnetic iron oxide nanoparticles (SPIONs), allow for concentrated PS drug delivery to invasive tumour sites, by utilising an external magnetic driving field force, which when applied directly above a tumour site causes magnetic NPs to aggregate and so rapidly intensifies PS uptake in this target region (i.e., physical targeting) [32,38]. Moreover, studies by Dang et al. (2017), have noted the use of manganese dioxide (MnO_2_) or perfluorocarbon (PFC) NPs to overcome the limitation of hypoxia against PDT, since they increase the oxygen levels in a TME when they decompose and so promote higher levels of ROS generation in target tumour sites, effectively enhancing PDT induced cell death [39]. Additionally, scintillating NPs are energy transducers which have the capability to convert X-rays into UV-visible photons and so through the utilisation of X-rays PDT PS deep seated tumour cell damage can be triggered, examples include the use of PS carrying upconversion nanoparticles (UCNPs) that can be excited by NIR light (e.g., 980 nm) and emit UV–visible light for enhanced therapeutic efficacy [40]. Lastly, metallic natured NPs, such a gold NPs, provide PDT with enhanced tumour destruction due to their photothermal properties, whereby they convert near-infra red light into heat and so provide multimodal cancer treatment opportunities [21,38].

## 7. Functionalised Nanoparticles for Effective and Active Targeted Photodynamic Therapy Tumour Selectivity Photosensitiser Drug Delivery

A tumour’s microenvironment (TME) is continuously changing and this complex behaviour plays an important role in cancer progression and PDT treatment, since a TME can hinder drug delivery systems and so render a treatment ineffective [24]. Thus, most NP PSs need to be constructed and functionalised according to the TME that they are targeting for a successful biophysiological interaction to occur, as to ensure effective PS drug uptake and retention for active indirect PS drug targeting [24,36]. 

**Figure 4:**
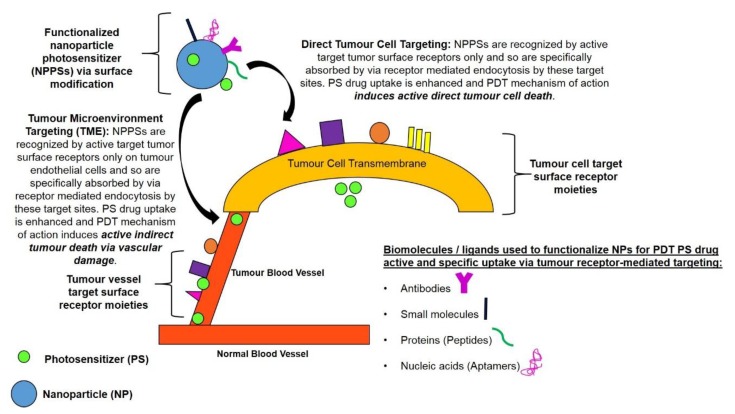
Diagram showing specifically active and targeted PS drug delivery to tumours via receptor mediated targeting. Tumour absorption of functionalised NPPSs is significantly increased via their surface modification with specific biomolecules or ligands. This active targeting mechanism takes advantage of highly specific interactions between biomolecules/ligands and certain TME tissues/blood vessels or cell surface antigens (receptor moieties) to increase cellular uptake and tumour retention of PS drugs. These active NPPSs drug systems enhance overall PS uptake in tumour cells only, which significantly enhances PDT induced tumour destruction, with the additional advantage that normal cells/tissues remain unaffected.

Tumour cell/tissue-specificity of PS drug delivery can be significantly increased via the surface modification of NPPSs to bind with targeting surface receptor moieties for active direct PS drug targeting [9,15]. The surface functionalisation of PS nano drug carrying systems with targeting receptor moieties which are overexpressed in tumour sites only, allows nano carriers to precisely recognise targeted tumours and so allow for active absorption and uptake of PS drugs in these specific cancer cells only (Figure 3 and Figure 4) [9,14,15]. This particular type of drug targeting system is known as the “magic bullet” or “smart drug delivery systems” in PDT cancer therapy [4,9]. The drug delivery systems consist mainly of two components, the first part of the system is able to recognise and bind to the target tumour site (providing precise drug transport) and the second part of the system being the actual PS drug itself, should be able provide effective PDT therapeutic action [4]. Thus, PDT active cancer nano-drug therapy targeting, implies the use of externally conjugated target moieties to a NP PS drug delivery system in order to enhance PS uptake and concentration in specific tumour cells [4].

Therefore, in order to functionalise nano-carrier particles to only target specific surface tumour receptor sites and so actively enhance PS drug uptake via RME and overall PDT, they are usually bound with specific targeting biomolecules/surface ligands [15]. These biomolecules or surface molecules for active tumour targeting include; antibodies, aptamers, peptides or small molecules that recognise tumour cell-specific or tumour associated antigens in the TME (Figure 4) [41,42]. Research has shown that this approach improves and concentrates PS drug localisation and active uptake in specific tumour cells only, while reducing the undesirable side effects of PS drugs to surrounding healthy tissues and unwanted phototoxicity [6,16]. Thus, for targeted and effective PDT, functionalised NPs are often used in research to efficiently incorporate and deliver hydrophobic PS drugs into only specific target tissues/cells, whereby via light activation they only produce ROS in tumorous tissues, destroying cancer cells only and leaving healthy tissues unharmed [16]. 

There are two approaches used for PDT PS drug tumour receptor-mediated targeting (Figure 4). Within the first approach the TME, such as the extracellular matrix or endothelial cell surface receptors, which are specifically expressed on tumours blood vessels, are targeted for enhanced PS drug delivery [3,28]. The second approach is to directly target tumour cell surface receptors for intracellular delivery of NPPS drugs [3,41]. Within this approach, NPPSs are functionalised to target the extracellular portion of tumour antigens that are overexpressed on the transmembrane of cancer cells and so PS drugs are taken up intracellularly specifically via RME [42].

### 7.1. Tumour Microenvironment, Tissue and Vascular Photosensitiser Nano-Drug Active Indirect Targeting

Angiogenesis is the synthesis of new blood vessels [43]. Since blood vessels in a TME provide an ample supply of nutrients and oxygen, their formation is vital in order to ensure a tumour survival, proliferation and metastatic spread [15,43]. Hence, selectively targeting PS nano-drug delivery to a TME (extracellular matrix, stroma, tissues or vascular nature), will enhance PDT ROS generation within these specific regions [20,43]. This in turn allows for direct damage to a tumour’s microvascular feed/blood vessels and so indirectly induces tumour destruction, due deprivation of nutrients and oxygen, enhancing the overall treatment efficacy of PDT [4,14,37]. 

Tumour vasculature endothelial targets such as vascular endothelial growth-factor receptors (VEGFRs), αvβ3 integrins, matrix metalloproteinase receptors (MMPs), collagen and vascular cell-adhesion molecule-1 (VCAM-1), have been exploited to achieve tumour-selective accumulation of PS nano-drug carriers in a tumour’s microvascular blood vessels [4,15]. Whereas, tumour associated immune cells (TAMS), tumour associated fibroblasts (e.g., FAP, α-SMA, FGFRs, tenascin-C and thrombospondin-1) and tumour initiating stem cells (e.g., CD133, EpCAM and aldehyde dehydrogenases), have been utilised to allow for NPPSs drug targeting within a TME [43].

However, this PDT mechanism of specific PS tumour vascular drug targeting remains debated, as some researchers argue that by excessively decreasing the vascular permeability of a tumour and its stroma, that this in turn sometimes causes hypoxia and decreased PS drug delivery [32,43]. It is better to account for enhanced tumour migration and metastasis, as well as PS drug resistance [43]. Therefore, TME NPPS drug targeting strategies need to be carefully designed, as to ensure tumour-inhibitory functions are targeted and not tumour-promoting functions [32]. Moreover, researchers tend to rather recommend using NPPS drug tumour direct cell targeting strategies in combination with TME and vascular drug targeting in order to ensure effective tumour destruction and enhanced PDT outcomes [4,43].

### 7.2. Tumour Cell Photosensitiser Nano-Drug Active Direct Targeting

In relation to functionalised NPs for specifically active PS drug delivery to selectively and directly destroy tumour cells and so indirectly enhance PDT, tumour cell transmembrane receptors can be directly targeted [4]. Tumour specific cell receptors which are usually exploited in targeting and delivering NPPS drugs directly into cancer cells are G-protein coupled receptors, integrins, folate receptors, transferrin receptors, ligand receptors (CD44), epidermal growth factor receptor (EGFR), fibroblast growth factors (FGFRs), sigma receptors, follicle stimulating hormone receptors, C-type lectin receptors, biotin receptors, and neuropilin receptors [4,17,21]. Depending on the intracellular location of the PSs, PDT ROS induces irreversible damage in target tumour cells, plasma membranes or vital subcellular organelles such as lysosomes, mitochondria, endoplasmic reticulum, Golgi apparatus or nucleus and so either apoptotic or necrotic forms cell death result in tumour destruction [14].

### 7.3. Types of Targeting Moieties for Active Tumour-Targeting Photosensitiser Nano-Drug Delivery Systems

In order to improve tumour selectivity and uptake of PS drugs via active targeting in either tumour cells or TME, NPPSs drug-carrying systems are often conjugated with specific targeting biomolecules or ligand moieties [15]. The moieties include monoclonal antibodies (mAb) and other proteins (such as transferrin), nucleic acids (aptamers), small molecules (folic acid), polymers (hyaluronic acid) and peptides (proteins), which are over-expressed on tumour cells only (Figure 4) [15,42]. These moieties have a specific affinity for receptors that are over-expressed on tumour cells and their vascular, but not normal cells and so facilitate effective PSs accumulation in tumour target sites only, increasing the efficacy of PDT with lessened collateral damage and unwanted side effects [17]. Recent research approaches (over the last 5 years) enhance NPPSs drug delivery using tumour-targeting moieties, and so increase the efficacy of PDT have been summarised in Table 2. 

Monoclonal antibodies (mAb) are a preferred class of targeting molecules for tumour cell receptor sites in order to enhance active and specific nano-PS drug delivery and so improve the cytotoxicity of PDT [32]. Some of the FDA approved mAb for targeted drug delivery in cancer cells include; Rituximab for the treatment of B-cell non-Hodgkin’s lymphoma, Trastuzumab for the treatment of human epidermal growth factor receptor 2 (HER2) expressing breast cancer, Bevacizumab for the treatment of vascular VEGFR expressing colorectal cancer, Cetuximab for the treatment of EGFR expressing colorectal cancer and head/neck cancer [4,43,44,45,46].

Research has noted that transferrin receptors (TfR) are over-expressed on tumour cells, due to their increased metabolic activity [47]. Moreover, the complex of transferrin-bound iron and transferrin receptor is a major route of cellular iron uptake via clathrin-coated pits to cellular endosomal compartments [47]. This membrane transferrin receptor-mediated endocytosis iron uptake pathway can be successfully exploited for the delivery of anti-tumour PS drugs, by functionalizing NPs which carry PS with transferrin proteins [4].

Aptamers are RNA or DNA nucleic acids capable of binding to target antigens with specificity conformations, which can correspond to antibodies [48,49]. They have many advantageous properties that include their small size, lack of immunogenicity in terms of provoking an immune response and ease of isolation [49]. Aptamers can be conjugated to PS drug delivery NPs to improve specific drug targeting delivery in tumour cells and so enhance PDT therapeutics [4,48]. The most successful and FDA approved aptamers for enhanced drug cancer drug delivery are those that are able to bind cancer cells which overexpress VEGFR proteins [9,14,48]. 

Hyaluronic acid (or hyaluronan) (HA) is a polysaccharide that is found within extracellular body fluids and is responsible for cellular growth, differentiation and migration in normal body cells [50]. However, HA is often found to be elevated in various types of tumour cancer cells and so gives them ability to invade and metastasise in other tissues [50]. Recently, researchers have started to investigate HA as a targeting moiety for NP PS enhanced drug delivery in PDT, since it can specifically bind to various cancer cells that over-express CD44 which is a HA tumour receptor [3,4,44,50].

Folic acid (FA) is an important Vitamin B in human body as it is the essential precursor for the synthesis of nucleic acids and some amino acids [51]. FA ligand is not produced by human cells and so requires cellular uptake via either receptor mediated endocytosis (RME) or carrier-based uptake mechanisms [51]. Within ovary, brain, kidney, breast, and lung malignancies it has been noted that these types of cancers tend to overexpress membrane bound folic acid receptors alpha (FR-α), since they have a high affinity for FA [51]. Thus, FA conjugation to nano-drug delivery systems has become a widely exploited strategy in order to enhance the specific cancer cell uptake of PS in targeted PDT applications [4,51].

Lastly, NPs carrying drugs can be functionalised with peptide or protein sequences, which target specific tumour cell surface receptors and so enhanced PS drug delivery [52]. Cell saturating and infusing peptides such as RGD are the most commonly targeted cancer moieties within enhanced drug delivery applications, as they bind strongly with αvβ3 integrins [52,53]. Moreover, with PDT nano-PS drug delivery studies, lipid NPs are among the most often utilised [4,15]. An alternative way for enhanced PS active uptake is to functionalise NP surfaces with certain short (approx. 30 amino acids) cell-penetrating peptides (CPPs) sequences which can pierce cell membranes and so transport PS drugs into cells or they can even be designed to directly target subcellular organelles [53]. 

## 8. Conclusions and Perspectives

PDT is unquestionably a highly effective and alternative therapeutic treatment for cancers. However, conventional PS drug delivery mechanisms have limitations in relation to poor solubility in physiological environments, adverse pharmacokinetics and poor tumour selectivity [14]. Surface functionalised tumour-targeting moieties NPPS drug delivery systems, as previously discussed can help overcome these limitations [14]. Biocompatible NPs have been developed to carry PS loads passively to tumour sites and their surfaces can be further functionalised and modified with targeting ligand moieties to make smart drug-delivery systems and so further actively augment the selective accumulation of PS loaded NPs at target tumour sites [4,14]. However, NP PS drug delivery design parameters need to be carefully considered to include precise surface functionalisation with appropriate targeting biomolecules (for tumour cells, tissues or TMEs) and PS-loading capacity, in order to eliminate non-specific PS toxicity in healthy tissues, as well as ensure specific and targeted PS uptake in target tumours only [3,11,36]. Moreover, once cellular uptake of the active nano drug carrier system has occurred, the rapid and responsive release of a PS needs to be considered as to ensure maximum ROS generation in targeted for effective tumour destruction [9,31,36]. Research by Ding et al. 2016 noted that stimuli-responsive NP carrier systems require further investigation, whereby PS drug biodistribution in response to a specific stimuli, being either external (variations in temperature, magnetic field, ultrasound intensity, light or electric pulses) or internal (changes in pH, enzyme concentration or redox gradients) can assist in controlled PS release from its nanocarrier platform to promote PDT efficacy [86].

In this review, we have evidenced the tremendous potential of specific receptor based photosynthetic nanocarrier platform drugs to actively promote the selective absorption of PS drugs in tumour sites only and so avoid unwanted side effects, as well as allow for the overall enhancement of PDT treatment for various cancers (Table 2). However, it must be noted that the research findings reported in Table 2 are in very early stages of in vitro research. Overall, active targeting alters the natural distribution patterns of a nano drug carrier molecule by directing it to a specific tumour organ, cell or organelle, whereas passive targeting relies on the natural distribution of a drug in a tumour via the EPR effect [53]. Either way, both processes within clinical environments rely on blood circulation and the location of the initial PS drug delivery for enhanced PDT and to date no actively targeted NP PS drug systems are commercially available [53]. Thus, researchers need to start further exploring and exploiting functionalised NPPSs for targeted PDT by performing more in vivo studies with more effective theoretical and mathematical models to allow for pre-clinical development and success during clinical trials [87,88]. The findings from these studies will allow for more conclusive results to be obtained in order to determine and further investigate if there are any other unforeseen limitations for this form of cancer treatment, in order to propel the application of targeted PDT PS drug delivery to the forefront of oncological interventions in the near future [89].

## Figures and Tables

**Table 1:** Composition-structure and properties of organic and inorganic nanoparticles.

Organic Nanoparticles	Type	Composition/Structure	Properties	Reference
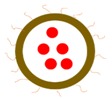	Lipids	Liposomes, micelles	Can carry hydrophobic drugs, controllable size	[5,9,20]
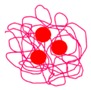	Polymeric	Poly(lactide-coglycolide) (PLGA), Glycerol, Chitosan, Co-Polymers, DNA monomers, hydrogels, dendrimers, cyclodextrins	Some biodegradable, high stability, small in size, biocompatible	[9,20,27,28]
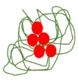	Protein/Peptide	Consist of simple peptides consisting of several amino acids e.g., Lactoferrin, albumin, gelatin, collagen, silk	Biocompatible, nonimmunogenicity, high tissue permeability,and rapid clearance from the body	[22,29,30,31]
**Inorganic Nanoparticles**	**Type**	**Composition/Structure**	**Properties**	**Reference**
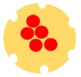	Ceramic/Silica	Spheres, shells, mesoporous	Biocompatible, allows for functionalisation, efficient loading of hydrophobic drugs, stable	[3,9,20]
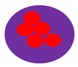	Quantum Dots	Cadmium selenide (CdSe), copper-indium-selenide (CISe), Cadmium telluride (CdTe)	Broad excitation, doesn’t photobleach, allows for functionalisation, high emission quantum yield, tuneable optics	[4,9,17]
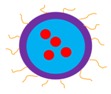	Magnetic	Iron oxide (SPIONS) or cobalt-based, usually sphere aggregates in dextran or silica	Paramagnetic or ferromagnetic	[9,32]
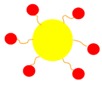	Metallic	Gold or silver spheres, rods, clusters, cadges or shells, as well as zinc and titanium dioxide	Biocompatible, theranostic (convert NIR to heat), allows for functionalisation, tuneable optics	[11,17,22,25,26,33,34]
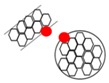	Carbon Based	Fullerene, carbon nanotube and graphene oxide	Biocompatible, allows for functionalisation	[9,21]

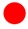
 **= Photosensitiser.**

**Table 2:** Targeting moiety approaches used to enhance NP PSs active drug delivery systems in tumours.

Targeting Moiety	Direct or Indirect Targeting	Tumour Overexpression Receptor	Cancer Cell Line or Tumour Model	Study Type	PS	NP	Results	Ref
mAB	Direct	erbB2 receptors	SK-BR-3 human breast cancer cells	In vitro	Porphyrin	Gold	Monophasic method NP PS elicited targeted PDT.	[54]
mAB	Direct	EGFR receptor	MKN45 human gastric cancer cells	In vitro	Curcumin	Chitosan/tripolyphosphate (TTP)	Enhanced uptake, superior PDT effect with a fourfold decrease in the IC_50_, however, PDT was limited to superficial tumours due to light penetration.	[55]
mAB	Direct	HER2 receptor	Breast carcinoma cell lines (SK-BR-3 & MDA-MB-231)	In vitro	Zinc phthalocyanine derivative (C11Pc)	PEG-Gold	Enhanced efficacy of PDT cell death when tumour-associated antigens were present on malignant cells.	[56]
mAB	Direct	EGFR receptor	CAL-27 oral squamous cell carcinoma (OSCC) & xenograft oral cancer tumour mouse model	In vitro & in vivo	Chlorin e6	Titanium dioxide (TiO_2_) PEG-up conversion	Enhanced intratumoural delivery, penetrated deep thick tumours with delayed tumour growth & 80% cell death.	[57]
mAB-Cetuximab	Direct	EGFR receptor	A431 squamous carcinoma cell line & xenografted mice	In vitro & in vivo	Chlorin e6	Methoxy poly(ethylene glycol)-b-poly(lactide) (mPEG-b-PLA) micelles	Enhanced uptake & effective PDT, at lowered doses tumour growth was inhibited by 84.8%.	[58]
mAB	Direct	Prostate-specific membrane antigen (PSMA-1)	Prostate cancer PC3pip cell line & xenografted mice	In vitro & in vivo	Silicon phthalocyanine PC 4	Gold	Nanodrug system enhanced uptake four fold, with significant cell death & tumours remained in remission 14 days post PDT.	[59]
mAB & Peptide	Direct	HER2 receptor or jacalin, a lectin specific for carbohydrate T anitigen	HT-29 colorectal adenocarcinoma cells & SK-BR-3 breast adenocarcinoma cells	In vitro	Zinc phthalocyanine photosensitiser (C11Pc)	PEG-Gold	Both T antigen & overexpressed HER-2 reported enhanced targeted PDT with 80–90% in HT-29 cells & >99% in SK-BR-3 cells.	[60]
Transferrin	Direct	Transferrin-receptor (TfR)	A549 human lung adenocarcinoma cell line & A549 tumour-bearing model	In vitro & in vivo	Hypocrellin A (HA)	Poly(d,l-Lactide-co-glycolide (PLGA) & carboxymethyl chitosan (CMC) nanoparticle	Selective uptake, apoptotic cancer cell death & significant tumour inhibition rate of 63% after target PDT treatment for 15 days in mouse models.	[61]
Transferrin	Direct	Transferrin-receptor (TfR)	Murine CT26 colon carcinoma cells & CT26 tumour-bearing mice.	In vitro & in vivo	IR780 iodide	Self-assembled transferrin-IR780	Notable targeting & tumour suppression in PDT cancer therapy.	[62]
DNA Aptamer	Direct	Specific targeting aptamers-TLS11a	HepG2 Hepatocellular carcinoma cell line xenograph mouse model	In vitro & in vivo	Chlorin e6	Gold	Programmable synergistic, targeted PDT, with hypoxia-activated chemotherapy treatment for hepatocellular carcinoma.	[63]
DNA G-quadruplex Aptamer	Direct	Sgc8 leukemia aptamer, which can specifically bind to protein tyrosine kinase 7 (PTK7) receptor	CEM cells CEM (CCL-119, T-cell line, human & Ramos (CRL-1596, B-cell line, human Burkitt’s lymphoma) & Cervical cancer (HeLa) mouse models	In vitro & in vivo	5, 10, 15, 20-tetrakis (1-methylpyridinium-4-yl) porphyrin (TMPyP4)	Zr-based nanoscale metal-organic frameworks (Zr-NMOFs)	Nanosystem induced 90% cell death of targeted cells & maintained more than 76% tumour inhibition within the entire experimental period.	[64]
DNA Aptamer	Direct	Sgc8 leukemia aptamer, which can specifically bind to protein tyrosine kinase 7 (PTK7) receptor	CEM (CCL-119, T-cell line, human & Ramos (CRL-1596, B-cell line, human Burkitt’s lymphoma)	In vitro & in vivo	Chlorin e6	Gold nanorod	Enhanced uptake & targeting, with notable PDT & photothermal cell destruction.	[65]
Hyaluronic acid	Direct & Indirect	CD44 ligands	Human colon HT29 cell line & murine tumour model	In vitro & in vivo	Chlorin e6	Hyaluronic acid conjugated to 5β-cholanic acid (5β-CA)	Effective biocompatibility, tumour targeting & suppression capacity. Tumour growth was significantly inhibited by 9.61 ± 1.09-fold.	[50]
Hyaluronic acid	Direct & Indirect	CD44 ligands	B16F10 melanoma cells in tumour model mice	In vivo	Chlorin e6	Carbon dot	Complete suppression of tumours & effective transdermal PDT of melanoma skin cancers	[66]
Folic acid	Direct	Folate receptor 1 (FOLR1)	A549 & SBC5 lung cancer cells & mouse lung orthotopic tumour models	In vitro & in vivo	Porphyrin	Porphyrin-lipid (porphysomes)	Only 24 to 28% of lung cancer cells noted to be viable after PDT treatment.	[67]
Folic acid	Direct	Folic acid receptor	Rat brain C6 glioma cancer cell line	In vitro	Spiropyran (SP)	Gold acrylic copolymer with imidazole groups	71.8% improved cellular uptake & enhanced tumour targeted PDT.	[68]
Folic acid	Direct	Folic acid receptor	KB oral cancer cell line	In vitro	Hematoporphyrin-stearylamine (HpSa)	Solid lipid (SLN)	Increased cellular uptake & enhanced PS phototoxicity.	[69]
Folic acid	Direct	Folic acid receptor	KB oral cancer cell line & murine xenograft model	In vitro & in vivo	Meta-tetra (hydroxyphenyl) chlorin (m-THPC)	Polymeric micelles	Reduced photosensitivity, with enhanced PDT & 92% tumour growth inhibition.	[70]
Folic acid	Direct	Folic acid receptor	Human cervical carcinoma (HeLa) cells	In vitro	Protoporphyrin IX (PpIX)	Gold	Enhanced drug delivery & phototoxic properties.	[71]
Folic acid	Direct	Folic acid receptor	Human breast MDA-MB-231 cancer cells	In vitro	Chlorin e6	Silica based	Enhanced uptake & PDT-induced mitochondrial damage & apoptotic cell death was observed.	[72]
Folic acid	Direct	Folic acid receptor	Human cervical carcinoma (HeLa) cells & tumour mouse model	In vitro & in vivo	Chlorin e6	Thermosensitive liposomes (TSL) with photothermal copper sulfide (CuS)	Enhanced uptake with controlled PS release, excellent phototoxicity & inhibited tumour growth.	[73]
Folic acid & DNA Aptamer	Direct & Indirect	C base-rich longer DNA would form C-quadruplex & folic acid binds to receptors	Human breast cancer MCF-7 cell line & tumour mouse model	In vitro & in vivo	Chlorin e6	Polyacrylic acid (PAA) coated upconversion nanoparticles (UCNPs)	Precise tumour targeting & efficient PDT with a switchable DNA/upconversion nanocomposite.	[74]
Folic acid & Peptide	Direct & Indirect	Folic acid receptor & cRGD targeting peptide to recognise αVβ3 integrin receptor	Human breast cancer MCF-7 cell line	In vitro	Chlorin e6 & Indocyanine green	Polymeric	Enhanced uptake & 85.9% tumour apoptosis.	[75]
Fibronectin mimetic peptide (Fmp)	Indirect	Integrin β1	Head & neck squamous carcinoma cell lines M4E, 686LN, & TU212 & murine xenograft model	In vitro & in vivo	Silicon phthalocyanine PC 4	Iron-Oxide	Enhanced uptake PDT efficacy with reduced PDT drug dose, showed nonspecific toxicity & greater inhibition of tumour growth than non-targeted drugs.	[76]
Cyclic peptide (c(RGDfc)	Indirect	Integrin αvβ3 receptor	UMUC3 human bladder cancer, Hela cells human cervical cancer & A549 human pulmonary carcinoma cell line & UMUC3 tumour mouse model	In vitro & in vivo	AIE luminogens (AIEgens) 2-((4-(2,2-bis(4 methoxyphenyl)-1-phenylvinyl) phenyl) (phenyl) methylene) malononitrile (TPE-red)	Aggregation-induced emission (AIE)	High tumour uptake efficacy with targeted PDT.	[77]
Peptide -Heptapepte (ATWLPP)	Indirect	Specific for the VEGF receptor, neuropilin-1 (NRP-1)	MDA-MB-231 breast cancer cells & rats bearing intracranial glioma	In vitro & in vivo	Chlorin e6	Silica based	Enhanced uptake, with effective interstitial PDT.	[78]
Peptide	Direct	Asialoglycoprotein receptor (ASGPR)	Human liver (HepG2) & Cervical (HeLa) cells	In vitro	Tetraphenylporphyrin tetrasulfonic acid hydrate (TPPS)	Pullulan-Functionalised Fe_3_O_4_ Nanoparticles with Mesopore Silica	Capable of targeting specific receptors, with efficient phototoxicity.	[79]
Peptide	Direct & Indirect	EGF peptide (YHWYGYTPQNVI-amide)	E29 rat glioma cancer cell line & tumour mouse model	In vitro & in vivo	Silicon phthalocyanine PC 4	PEG-Gold	Drug conjugate enhanced PS delivery, as well as enhanced PDT therapeutic efficacy two-fold.	[80]
Peptide	Indirect	Cationic diphenylalanine (H-Phe-Phe-NH2·HCl, CDP)	MCF-7 breast cancer & tumour bearing mice	In vitro & in vivo	Chlorin e6	Cationic dipeptide	Enhance drug targeting & uptake, with PS controlled release & almost complete tumour eradication.	[29]
Peptide	Indirect	Fluorenylmethoxycarbonyl-l-histidine (Fmoc-H), & *N*-benzyloxycarbonyl-Lhistidine-l-phenylalanine (Z-HF)	MCF-7 breast cancer & tumour bearing mice	In vitro & in vivo	Chlorin e6	Metallo Fmoc-H/Zn^2+^ & Z-HF/Zn^2+^	Desirable stability & smart responsiveness, with enhanced Chlorin e6 internalization.	[30]
Peptide	Indirect	Cationic dipeptide (H-Phe-Phe-NH_2_·HCl, CDP)	MCF-7 breast cancer	In vitro	Rose Bengal (RB)	Cationic dipeptide	Biocompatible, with improved tissue uptake & induced serious two-photon toxicity.	[31]
Peptide	Indirect	Neuropiline-1 (KDKPPR)	Human umbilical vein endothelial cells (HUVEC) & skinfold chamber model in mice	In vitro & in vivo	5-(4-carboxy phenyl)-10,15,20-triphenylporphyrin	Silica-based (AguIX)	Enhanced uptake, with improved PDT photoxic effect.	[81]
Peptide	Indirect	F3 peptides	GS-9L & F98 rat glioma & MDA-MB-435 human breast carcinoma cell lines	In vitro	Methylene blue	Polyacrylamide	Enhanced targeting with excellent PDT efficacy increasing with dose & irradiation time.	[82]
Peptide	Direct	Cationic cell-penetrating peptides Tat (48–57)	KB human oral epidermoid carcinoma & MC28 methylcholanthrene-induced rat fbrosarcoma cell lines		Chlorin e6	Cationic dipeptide	Enhanced endosomal membrane targeting with high photodynamic efficacy.	[83]
Peptide-Lactose	Direct & Indirect	Galectin-1 receptor	Human breast MCF-7 cell line	In vitro	Zinc phthalocyanine	Gold	Enhanced uptake, excellent ROS generation & efficient PDT.	[84]
Magnetic field targeting	Physical external magnetic force targeting		Human breast MCF-7 cell line	In vitro	Meso-tetrakis (4-hydroxyphenyl) porphyrin	PEGylated gold SPIONs	Enhanced PS uptake was noted & after PDT treatment 79% cell death was reported.	[32]
Magnetic field targeting	Physical external magnetic force targeting		SW480 colon carcinoma cells & athymic mouse model	In vitro & in vivo	8 2,7,12,18-Tetramethyl-3,8-di(1-propoxyethyl) -13,17-bis-(3-hydroxypropyl)porphyrin (PHPP)	Magnetic Fe_3_O_4_ chitosan	Excellent targeting & uptake, non-toxicity & high photodynamic efficacy.	[85]
